# Serums and Vaccines in Influenza

**Published:** 1919

**Authors:** 


***Serums and Vaccines in Influenza.*** EDITORIAL. *The Journal of the American Medical Association,* October, 26, 1918.

There is not as yet any specific serum or other specific means for the cure of influenza, and no specific vaccine for its prevention. All efforts for treatment and prevention now undertaken are simply experiments, and the true value of the results cannot be predicted. The only noteworthy new method so far is the injection, in severe cases of influenzal pneumonia, of the serum of patients who have recovered from such pneumonia; analogous procedures have given good results in scarlet fever and other diseases, and those reported in influenzal pneumonia appear promising.

Two kinds of vaccines are used in the hope that they may have preventive effects. One consists solely of killed influenza bacilli; the other is a mixed vaccine of the most important bacteria in the respiratory tract in influenza, pneumococci, streptococci and influenza bacilli. There is, however, no basis on which promises of protection from vaccines may be made; they may be harmless, and they may or may not be of preventive value.

